# Catching the Silent Culprits: *TERT* Promoter Mutation Screening of Minimally Invasive Follicular and Oncocytic Thyroid Carcinoma in Clinical Practice

**DOI:** 10.1007/s12022-024-09828-x

**Published:** 2024-10-04

**Authors:** L. Samuel Hellgren, Adam Stenman, Kenbugul Jatta, Vincenzo Condello, Catharina Larsson, Jan Zedenius, C. Christofer Juhlin

**Affiliations:** 1 Department of Oncology-Pathology, Karolinska Institutet, Stockholm, Sweden; 2 Department of Pathology and Cancer Diagnostics, Karolinska University Hospital, Stockholm, Sweden; 3 Department of Molecular Medicine and Surgery, Karolinska Institutet, Stockholm, Sweden; 4 Department of Breast, Endocrine Tumors, and Sarcoma, Karolinska University Hospital, Stockholm, Sweden

**Keywords:** Follicular thyroid carcinoma, Oncocytic thyroid carcinoma, Minimally invasive, *TERT* promoter mutation

## Abstract

De-escalation of thyroid cancer treatment is crucial to prevent overtreatment of indolent disease, but it remains important to identify clinically aggressive cases. *TERT* promoter mutations are molecular events frequently associated with high-risk thyroid tumors with poor outcomes and may identify cases at risk of dissemination. In various international guidelines, small minimally invasive follicular thyroid carcinoma and oncocytic thyroid carcinoma (miFTC/miOTC) are classified as low-risk lesions and are not recommended adjuvant treatment. Our study aimed to explore the association between size-based risk assessment and *TERT* promoter mutations. Between 2019 and May 2024, 84 miFTCs/miOTCs diagnosed at our department underwent digital droplet PCR analysis targeting *TERT* promoter mutational hotspots C228T and C250T in clinical routine. *TERT* promoter mutations were found in 10 out of 84 cases (11.9%). Mutated cases were pT1 (*n* = 1), pT2 (*n* = 3), or pT3 (*n* = 6). Patients with mutated tumors were older compared to patients with wild-type tumors (median age of 71 years vs. 57 years, *p* = 0.041). There were no significant differences regarding patient sex, tumor size, Ki-67 labeling index, or the presence of distant metastases. Notably, 30% of mutations displayed variant allele frequencies < 10%, possibly suggesting subclonal events. To conclude, *TERT* promoter mutations in miFTCs and miOTCs were associated with higher patient age and were often suspected to be subclonal. However, they did not affect clinical outcomes, possibly due to short follow-up. Reflex testing for this genetic alteration in miFTCs and miOTCs could be justified regardless of tumor size, though the clinical benefit remains uncertain.

## Introduction

Follicular thyroid tumors represent the most common neoplasms found in the thyroid gland. The disease spectrum encompasses both benign follicular thyroid adenomas (FTAs) and malignant follicular thyroid carcinomas (FTCs). Diagnostic criteria rely on histomorphological features, wherein FTCs exhibit malignant characteristics such as capsular and/or vascular invasion, whereas FTAs lack both. However, follicular tumors pose a challenge due to the difficulty in distinguishing these malignant features. From a prognostic perspective, FTCs and oncocytic thyroid carcinomas (OTCs) are further categorized into minimally invasive (mi), encapsulated angioinvasive (eai), and widely invasive (wi) subtypes, demonstrating clear associations with patient outcomes [1]. Additionally, the proliferation marker Ki-67 has been identified as a potential prognostic tool of value to identify clinically aggressive cases [2]. Also, genetic studies have demonstrated superior resolution in identifying cases with poor prognosis through molecular approaches using tumor mutational burden analyses, although these analyses are cumbersome and have not yet been widely implemented in the clinical setting [3]. Hence, there is a growing demand for additional diagnostic and prognostic tools that are clinically straightforward. Among several markers previously examined, the presence of a mutation in the *telomerase reverse transcriptase* (*TERT*) promoter region has been found particularly promising [4]. *TERT* promoter mutations occur as two hotspot mutations, C228T and C250T, which lead to increased *TERT* activity and telomerase activation. Telomerase activation leads to telomere lengthening, which facilitates the immortalization of cancer cells. *TERT* promoter mutations have been related to a higher rate of recurrence and mortality in thyroid carcinomas [1, 5–7]. However, not all *TERT* promoter-mutated carcinomas result in worse outcomes than wild-type cases. This variability reflects the heterogeneous nature of thyroid cancer and the influence of other co-variables, such as the presence of additional genetic mutations, tumor subtype, and patient characteristics, that can also impact disease outcomes. About 15–20% of all FTCs harbor a *TERT* promoter mutation [4, 8, 9], and mutated tumors are often associated with a worse prognosis compared to wild-type tumors. In contrast, *TERT* promoter mutations are only rarely described in FTAs [10–12]. Previous studies have examined the prognostic and diagnostic value of *TERT* promoter mutations in follicular thyroid tumors. In one study, a group of thyroid tumors was examined for *TERT* promoter mutations preoperatively on fine needle aspiration cytology material and *TERT* mutations were only found in malignant tumors [13], illustrating its diagnostic potential. Similar findings have been reported by others, but occasional cases of *TERT* promoter mutations in benign entities may reduce the specificity of the analysis in pre-operative material [14, 15]. Subsets of FTAs and follicular tumors of uncertain malignant potential (FT-UMPs) with a *TERT* promoter mutation may recur with metastases, while tumors without the mutation usually do not [10, 16], illustrating its prognostic potential. A similar association seems to be true for FTCs, as *TERT* promoter mutations may detect patients at risk of a poorer outcome [17, 18].

Among the histological subtypes of FTC, miFTC stands out as the most common and least aggressive, often categorized as an indolent tumor [19, 20]. Several guidelines, including the American Thyroid Association management guidelines, refrain from recommending adjuvant treatment for patients with small miFTCs due to their classification as low-risk tumors [20]. However, despite being classified as low-risk tumors, it remains unclear to what extent they harbor *TERT* promoter mutations. Moreover, there is limited research on whether *TERT* promoter-mutated miFTCs/miOTCs exhibit a higher metastatic rate than wild-type tumors or how the mutational status should influence the treatment approach for these tumors. This study aims to evaluate a prospective screening program that assesses the *TERT* promoter mutational status in miFTCs/miOTCs and to determine if such occurrences should impact their management.

## Materials and Methods

### Study Design

We introduced a prospective screening program to examine to what extent *TERT* promoter mutations are present in miFTCs/miOTCs. Included tumors were reflex tested for *TERT* promoter mutations using digital droplet PCR (ddPCR), which has a higher sensitivity compared to Sanger sequencing [21]. The analysis was performed in clinical routine as part of the normal diagnostic work-up of these tumors, and the results were included in the pathology report. We included all patients with a miFTC/miOTC diagnosed at Karolinska University Hospital, Stockholm, Sweden, between 2019 and May 2024. Other thyroid tumor types, including other subtypes of FTCs, were excluded. Areas with the slightest possibility of venous invasion were analyzed for CD31 and CD61 immunohistochemistry, to safely rule out vascular infiltration in our cohort. Histopathological, immunohistochemical, and clinical variables were collected at the end of the study (May 2024). Small subsets of cases have been previously assessed for *RAS* hotspot mutational status covering exons 12/13 and/or 61 using DNA sequencing techniques [22, 23], which allowed us to incorporate this data.

### Histopathological and Clinical Information

All tumors were diagnosed by subspecialized endocrine pathologists in a tertiary thyroid cancer center. Tumors were examined on microscopy slides, stained with hematoxylin and eosin. All histopathological data were collected from the pathology reports at the end of the study. Parameters such as extrathyroidal extension, tumor size, pathological tumor stage, evidence of disease recurrence, and distant metastasis were collected. Clinical data were extracted from the patients’ medical charts, including sex, age, type of surgery, adjuvant treatment, clinical signs of metastasis, and cause of death when applicable. Disease recurrence was evaluated using a combination of imaging techniques and laboratory tests. We utilized CT scans, scintigraphy, ultrasound, and serum thyroglobulin levels in relevant cases to monitor for recurrence. Tumors with metastatic disease were re-evaluated by the authors LSH and CCJ as a precautionary measure to ensure that the tumors were correctly classified as miFTC or miOTC, and that no foci of angioinvasion were missed. An ethical approval (approval number 2015–959-31) was obtained from the Swedish Ethical Review Authority.

### Histological Definitions

The tumors were included during a period in which an updated version of the WHO classification of endocrine and neuroendocrine tumors was introduced. Tumors diagnosed between 2019 and the beginning of 2022 were diagnosed according to the criteria of the 2017 WHO classification, while tumors diagnosed during the latter part of 2022 until the end of inclusion were diagnosed according to the criteria of the 2022 WHO classification. However, the definition of miFTC/miOTC has not changed between WHO editions.

**Fig. 1:**
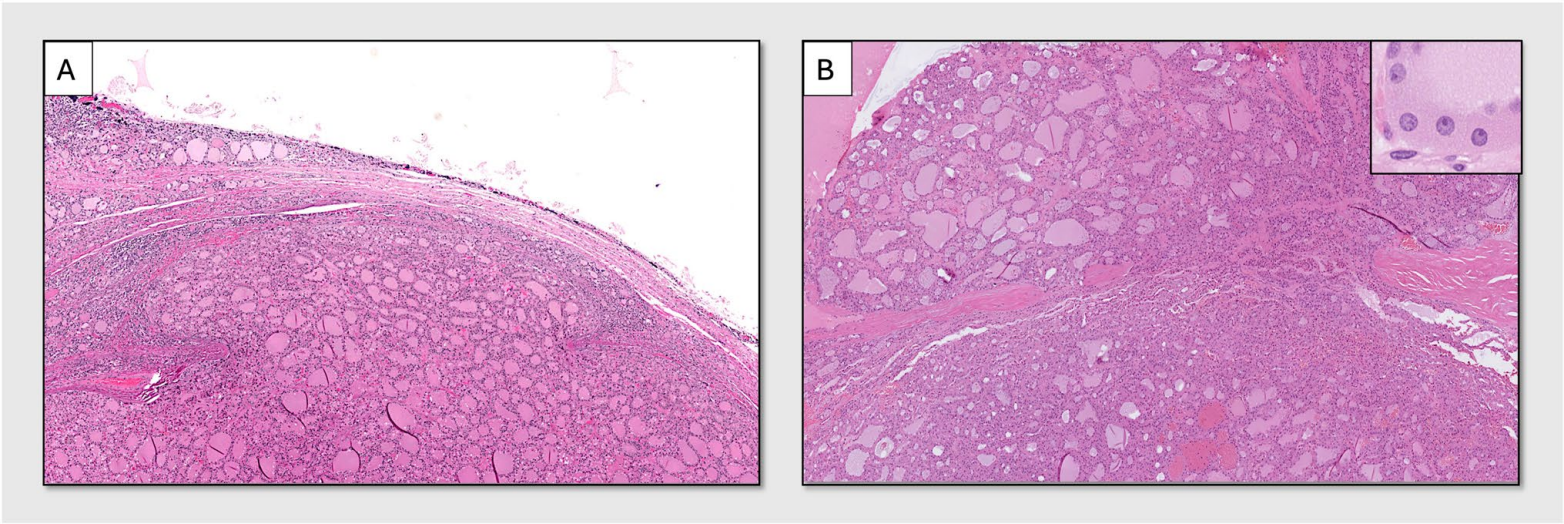
Hematoxylin and eosin stained minimally invasive follicular thyroid carcinoma (miFTC) and oncocytic carcinoma (miOTC). **A** MiFTC with a *TERT* promoter mutation. Note the mushroom-like protrusion of tumor cells through the capsule. No areas with vascular invasion were noted. **B** MiOTC without a *TERT* promoter mutation, depicting the site of capsular invasion. In the top right corner of the image, a higher magnification of the tumor is inserted to illustrate the oncocytic tumor cells

The diagnosis of FTC is characterized by well-differentiated follicular cell-derived tumors with invasive features, without the nuclear features of papillary thyroid carcinoma (PTC). FTCs with more than 75% oncocytic tumor cells are regarded as OTCs. The FTCs and OTCs are divided into three subtypes based on the presence and extent of capsular and vascular invasion. The miFTC/miOTC subtype, which is the focus of this study, is characterized by limited capsular invasion and no vascular invasion. Capsular invasion was defined as when tumor cells invade through the entire thickness of the surrounding tumor capsule (as illustrated in Fig. 1). Vascular invasion was defined as the invasion of vessels within or beyond the tumor capsule, with intravascular tumor cells attached to the vessel wall or mixed with fibrin. Equivocal cases were assessed using CD31 and CD61 immunohistochemistry.

The diagnosis of differentiated high-grade thyroid carcinoma (DHGTC) was introduced in the 2022 WHO classification system [1]. This tumor is defined by a well-differentiated follicular cell-derived thyroid malignancy (PTC or FTC), that exhibits high-grade features such as tumor necrosis and/or a mitotic count of ≥ 5 mitoses per 2 mm^2^ [1]. In our study, we reviewed the pathology reports of all included cases and annotated high-grade features (mitoses and necrosis) in miFTCs/miOTCs that were diagnosed according to the pre-DHGTC era using the 2017 WHO classification system. Using this approach, any miFTC/miOTC in our study cohort that fulfilled the DHGTC criteria was included in the study.

Tumors of all stages were included. Tumor stages were determined according to the 8th edition of the American Joint Committee on Cancer (AJCC) staging manual. All cases that were metastatic on follow-up were re-evaluated microscopically by two of the authors (LSH and CCJ) to verify the miFTC/miOTC diagnosis and to firmly exclude vascular invasion. As part of standard routine practice in our department, FTCs and OTCs are typically fully embedded, with the capsule often examined at multiple levels.

### Telomerase Reverse Transcriptase (*TERT*) Promoter Mutational Analysis

*TERT* promoter mutation analyses were performed using ddPCR. The analysis was performed as a part of clinical routine testing. Analyses were performed on DNA from formalin-fixed paraffin-embedded (FFPE) material from a representative tissue slide chosen by the pathologist. When selecting representative sections, care was taken so that only the miFTC/miOTC lesions were included. If an adjacent PTC was identified, slides containing this separate tumor were not selected for genetic analysis. The DNA extraction and ddPCR analysis were performed using the same methodology as previously published [21]. Mutation-positive and mutation-negative references from commercially available male genomic DNA were used as internal controls. Tumors were considered mutated if the analysis showed a variant allele frequency (VAF) > 1%. With a negative test result, the tumors were considered wild-type tumors. The test result was included in the pathology report, and all cases were subsequently discussed at weekly tumor board meetings at the Karolinska University Hospital, Stockholm, Sweden.

### Statistical Analyses

All statistical analyses were performed using IBM SPSS Statistics version 29 (SPSS Inc, Chicago, IL, USA). The *TERT* promoter mutated tumors were compared to *TERT* promoter wild-type tumors regarding clinical and histopathological parameters. Fisher’s exact test was used to compare categorical variables and the Mann–Whitney *U* test was used to analyze and compare continuous variables. *P* values < 0.05 were regarded as statistically significant.

## Results

### General Characteristics of the Study Cohort

During the study period, a total of 895 well-differentiated thyroid carcinomas were operated on and diagnosed by histopathology at our department, including 714 primary PTCs, 128 primary FTCs, and 53 primary OTCs. After inclusion, 84 patients with 84 primary thyroid tumors were included in the study, of which 25 (33.8%) were miOTCs and 59 (66.2%) were miFTCs. Of these tumors, 10 (11.9%) harbored a *TERT* promoter mutation. The mean follow-up time of the cohort was 21.7 months, ranging from 0 to 53 months. Patients had a mean age of 55.7 years and a mean tumor size of 39.5 mm. Histopathological data and patient characteristics of all included patients, divided according to *TERT* promoter mutational status, are summarized in Table 1. In short, patients with *TERT* promoter mutated tumors were older compared to patients with wild-type tumors (median age of 71 years vs. 57 years, *p* = 0.041). The mutated tumors received more radioactive iodine (RAI) treatment compared with wild-type tumors (*p* = 0.034). No significant differences were found between these groups regarding other variables examined (Table 1). Previously published *RAS* genotypes (covering codons 12/13 and/or 61) were available for nine cases, of which six were mutated (67%) and three (33%) were wild-type. The mutated cases were *HRAS* Q61R (*n* = 3), *NRAS* Q61R (*n* = 1), *NRAS* Q61K (*n* = 1), and *KRAS* Q61R (*n* = 1). Of these, only one case had a synchronous *TERT* promoter mutation (case no. 23).

**Table 1:** Histopathological and clinical data of all included patients

	*TERT* mutated	*TERT* wild-type	*P* value
Number of patients, *n*	10	74	
Sex, female, *n* (%)	6 (60)	59 (79.7)	0.223
Age at surgery, median (range)	71 (43–76)	57 (19–86)	0.041*
Tumor size, mm, median (range)	45 (17–100)	32.5 (9–130)	0.122
Ki-67 labeling index, median (range)	5.8 (1–12)	3.9 (1–14.7)	0.117
miOTC, *n* (%)	2 (20)	23 (31.1)	0.716
miFTC, *n* (%)	8 (80)	51 (68.9)	
Tumors with DHGTC features, *n* (%)	0 (0)	4 (5.4)	1
pT1, *n* (%)	1 (10)	10 (13.5)	
pT2, *n* (%)	3 (30)	38 (51.4)	
pT3, *n* (%)	6 (60)	26 (35.1)	
pT4, *n* (%)	0 (0)	0 (0)	
Extrathyroidal extension, *n* (%)	0 (0)	0 (0)	
RAI dose, median (range)	3.7 (1.1–3.7)	1.1 (0–5.4)	0.034*
Metastasis, *n* (%)	0 (0)	3 (4.1)	1
Dead, *n* (%)	1 (10)	1 (1.4)	0.225

*TERT* telomerase reverse transcriptase, *pT1-4* pathological tumor stage according to American Joint Committee on Cancer (AJCC) Cancer staging manual 8th edition, *miOTC* minimally invasive oncocytic thyroid carcinoma, *miFTC* minimally invasive follicular thyroid carcinoma, *DHGTC* differentiated high-grade thyroid carcinoma, *RAI* radioactive iodine. *n* sample size. *P values < 0.05 were considered statistically significant. Data in bold emphasis indicate significant *p* values

### *TERT* Promoter Mutations in Different Tumor Stages

In our study, 11 patients (13.1%) had a tumor measuring less than 20 mm. Of these pT1 tumors, one (9.1%) was *TERT* promoter mutated. In pT2 tumors, 3 out of 41 patients (7.3%) were *TERT* promoter mutated, and in pT3 tumors, 6 out of 32 patients (18.8%) were *TERT* promoter mutated. No tumor was classified as pT4. When looking at the treatment given, the multidisciplinary team meeting decided to deviate from the current guidelines in two cases with *TERT* promoter mutation by giving a higher RAI dose than recommended. These tumors measured 37 mm and 38 mm, respectively, and were therefore close to the threshold for the pT3 tumor stage. None of the tumors with a *TERT* promoter mutation would have been classified as a DHGTC according to the 2022 WHO classification. Detailed information about the mutated tumors is shown in Table 2.

**Table 2:** Histopathological and clinical data of tumors with *TERT* promoter mutation

Case ID	Age at surgery	Sex	Diagnosis	Size (mm)	Ki-67 index (%)	*TERT* mutation	Tumor cell content (%)	VAF (%)	Metastasis	Follow-up time (months)
6	69	Female	miFTC	45	6.5	C228T	70	37	No	51
8	74	Female	miFTC	23	12	C228T	80	6	No	41
11	74	Male	miOTC	50	5.1	C228T	70	19	No	45
15	74	Male	miOTC	50	2.1	C228T	80	16	No	44
23	76	Female	miFTC	80	8.7	C228T	90	47	No	25
26	52	Male	miFTC	37	10.4	C228T	80	54	No	32
50	43	Female	miFTC	45	1	C228T	80	< 10	No	17
51	67	Female	miFTC	38	4	C250T	90	89	No	20
56	62	Male	miFTC	100	3.5	C228T	80	26.1	No	14
84	73	Female	miFTC	17	11.8	C288T	70	1.3	No	1

*TERT* telomerase reverse transcriptase, *VAF* variant allele frequency, *miOTC* minimally invasive oncocytic thyroid carcinoma, *miFTC* minimally invasive follicular thyroid carcinoma

### Variant Allele Frequencies of *TERT* Promoter Mutations

The use of ddPCR allows us to obtain specific VAFs for each mutation found. In the cohort, tumor cell content in all representativity sections from each case was always ≥ 70%, sometimes as high as 90%. Therefore, the VAF of the *TERT* promoter mutations could in theory provide insights regarding the clonal status of this mutation in each tumor, potentially allowing comparisons between the cases. The VAFs ranged from 1.3 to 89% (Table 2), with an average VAF of 30.4% (in which one case determined as “ < 10%” was assumed to reach 9%). Subsets of cases with very low VAFs were re-analyzed with similar results, including the case with a VAF of 1.3%.

### Patients with Metastatic Disease

Patient characteristics of the patients with regional or distant metastatic disease are summarized in Table 3. The bone metastases in patients 9 and 25 were confirmed through postoperative scintigraphy and elevated thyroglobulin levels and were detected 3 months and 4 months after surgery, respectively. The lymph node metastasis in patient 37 was identified during the histological workup following surgery. All three patients with metastatic disease had a *TERT* promoter wild-type tumor. Patients with metastasis had a mean age of 52 years (range 43–59), a mean tumor size of 45.7 mm (range 12–70 mm), and a mean Ki-67 labeling index of 5.4% (range 2.3–11%). None of the patients with metastasis had a tumor that would have been classified as a DHGTC according to the 2022 WHO classification. One of the tumors with metastasis was a pT1 tumor, measuring 12 mm, representing a metastatic rate of 9.1% of all pT1 tumors. This pT1 tumor was identified with metastatic disease to a lymph node at the time of diagnosis and has been previously published [23]. As a part of the routine diagnostic procedure, this tumor was analyzed with a more extensive DNA sequencing analysis using next-generation sequencing (NGS). The analysis showed that the tumor harbored an *NRAS* codon 61 mutation, but no other coding alterations were covered by our clinical panel. All miFTCs/miOTCs with distant metastases were re-evaluated from a histological standpoint, but no cases were shown to exhibit vascular invasion. Two patients died during follow-up, one of malignant pleural mesothelioma and one of COVID-19. No patients died of miFTC/miOTC.

**Table 3:** Histopathological and clinical data of tumors with metastasis

Case ID	Age at surgery		Sex	Diagnosis	Size (mm)	Ki-67 index (%)	*TERT* mutated	Metastatic location	Metastasis detected at	Follow-up time (months)
9	59	Female		miFTC	55	11	No	Bone	Follow-up	47
25	54	Female		miFTC	70	2.3	No	Bone	Follow-up	34
37	43	Female		miFTC	12	3	No	Lymph node	Diagnosis	28

*TERT* telomerase reverse transcriptase, *miFTC* minimally invasive follicular thyroid carcinoma

## Discussion

In this study, we evaluated a prospective screening program that analyzed miFTC/miOTC tumors for *TERT* promoter mutations. We included 84 patients with an overall *TERT* promoter mutational frequency of 11.9%. This frequency is lower than in previous study cohorts containing all FTC subtypes, ranging between 15 and 20% [4, 6, 8, 9, 17, 24, 25]. The frequency is, however, similar compared to other study cohorts that have described the frequency of *TERT* mutations in the miFTC subtype alone, ranging between 9.7 and 10.3% [8, 17].

In contrast to most other studies on this topic, we opted for ddPCR as the method of choice in our screening program. This highly sensitive technique enables the detection of mutations with VAFs < 10%. This stands in clear contrast to conventional Sanger sequencing, which typically cannot detect mutations occurring in less than 10% of the interrogated DNA [21]. Interestingly, three out of ten (30%) tumors with *TERT* promoter mutations exhibited VAFs < 10%, thus arguing that a substantial subset of these tumors potentially may exhibit subclonal mutations not easily detected by conventional Sanger. Similar findings in FT-UMPs have been reported by us earlier [21]. As no metastatic events were recorded for any miFTC/miOTC with mutations in our series, the value of detecting this aberrancy in a fraction of tumor cells remains unclear. However, it is evident that ddPCR increases the likelihood of detecting this high-risk molecular aberration compared to traditional Sanger sequencing. As the objective of this study was to specifically target a single, high-risk genetic event, we did not use next-generation sequencing (NGS) to screen for a broader range of genetic alterations. While NGS analyses may reveal additional genetic events of interest, they are not yet of universal use in clinical routine at our department, which is guided by overall cost–benefit considerations and the long turnaround time.

In our cohort, one of the eleven miFTC/miOTC tumors measuring less than 20 mm harbored a potential subclonal *TERT* promoter mutation with a VAF of 1.3%. This patient did not exhibit metastases, and therefore, it is not known if the presence of this very low-abundant *TERT* promoter variant might affect the risk of metastases in the future. The finding of a potentially subclonal *TERT* promoter variant in a pT1 tumor may not necessitate the need to diverge from existing guidelines, as the tumor is still considered low-risk and not in need of further treatment. However, it is still intriguing to find a fraction of tumor cells with a high-risk genetic variant in such a clinically indolent case. In fact, our national guidelines would leave room for a more personalized follow-up approach for this individual patient, which would involve carefully discussing the pros and cons of total thyroidectomy and RAI ablation with the patient. If the patient is treated with lobectomy alone, we will monitor them through clinical examinations and imaging (ultrasound, CT scans, etc.) as appropriate. Although thyroglobulin measurements are unreliable in this context, a significant increase over time could suggest recurrent disease and may warrant consideration. As mentioned above, the importance of these subclonal mutations is elusive, and since no mutated tumors were diagnosed with metastases, it is uncertain if *TERT* promoter mutations have the same significance in miFTCs/miOTCs as in other FTC subtypes. Therefore, caution is necessary to avoid overestimating the risk until the role of fractional *TERT* promoter mutations in thyroid neoplasia has been characterized.

The overall metastatic rate in our series was 3.6%, which is lower than the 17.9% published previously in a mixed cohort of all FTC subtypes [2]. In our small series, *TERT* promoter mutations were not associated with metastatic disease in this study since none of the metastatic tumors had a *TERT* promoter mutation. This is not entirely expected, since the presence of mutation has repeatedly been shown to be a poor prognostic factor in previous studies of other subtypes of thyroid tumors—but this observation could also merely mirror our limited sample size and limited follow-up time. One of the *TERT* promoter wild-type tumors measuring less than 20 mm exhibited metastasis already at the time of diagnosis. This tumor harbored an *NRAS* codon 61 mutation, which has been associated with a higher rate of metastases compared to other *RAS* mutations in FTCs [26]. In the small cohort of pT1 tumors, the metastatic rate was 9.1%. This is a similar rate compared to a pT1 group with a rate of 12.1% that includes all FTC subtypes [2]. Although small miFTCs are typically regarded as low-risk, a fraction of cases can still present with metastasis at the time of diagnosis. Interestingly, the patient with a pT1 tumor presenting with metastatic disease did not display any known risk factors for aggressive clinical behavior according to current clinical risk stratification. Unfortunately, *TERT* promoter mutational analysis did not provide insights in this regard. As no miFTCs with distant metastases were found to exhibit vascular invasion upon histological re-evaluation, one must acknowledge a small but biologically relevant risk of dissemination even in these tumors with only limited capsular invasion and no *TERT* promoter mutation.

We must emphasize the limitations of our study, particularly the small sample size and short follow-up period, which partially restrict our ability to draw definitive conclusions regarding *TERT* promoter genotypes and their impact on patient outcomes. However, given that previous retrospective analyses of large thyroid cancer cohorts have established *TERT* promoter mutations as a high-risk event linked to poor prognosis, we instead aimed to conduct a prospective study to assess the real-world outcomes of a positive mutational finding in indolent tumor types like miFTC and miOTC. Our results are further complicated by the presence of low VAFs in some tumors, possibly indicating subclonal mutations of uncertain clinical significance.

In our series, *TERT* promoter mutations were associated with older age, which is consistent with previous studies [25]. Surprisingly, other known risk factors for a *TERT* promoter mutation did not differ between the groups with mutated or wild-type tumors. Usually, *TERT* promoter mutations are restricted to larger thyroid tumors in older patients. In this study, the tumor size difference between the mutated and wild-type groups did not reach statistical significance. This may be attributed, at least in part, to the discovery of a 17 mm tumor with a subclonal *TERT* promoter mutation, which, to our knowledge, represents one of the smallest miFTC/miOTC with a *TERT* promoter mutation to date. Further investigations using the more sensitive ddPCR methodology could potentially uncover whether subclonal mutations are more prevalent in smaller tumors and younger patients than previously believed and whether this phenomenon carries any clinical significance.

Since *TERT* promoter mutations were more frequent in larger tumors and rare in the smallest ones, the screening program was most valuable for larger tumors. Although the mutated tumors did not exhibit any metastases, the relatively short follow-up and small sample size may affect the results. However, it is clear from this study that the presence of a *TERT* promoter mutation does not have the same impact in this FTC subtype compared to a mixed study cohort including all subtypes. In a previous study [17], the authors analyzed all three subtypes of FTC separately for *TERT* promoter mutations. They found that the presence of *TERT* promoter mutations did not affect the prognosis in miFTC, while it was of clinical importance in eaiFTC and wiFTC. As *TERT* promoter mutations did not impact the prognosis of our miFTC/miOTC patients, other tumor groups might be more suitable for mutational screening, such as larger tumors with vascular invasion. Future studies are required to draw more definitive conclusions.

We conclude that a proportion of miFTCs/miOTCs carry *TERT* promoter mutations that may be subclonal, and the mutation is associated with a higher patient age. Although mutations are rare in small tumors, we discovered a mutated tumor among those measuring less than 20 mm. Although our molecular risk stratification cannot be stated to contradict the current approach of releasing small, low-risk lesions as outpatients without additional treatment, the finding of single pT1 tumors with potential, low-abundance mutations suggests that *TERT* promoter mutational testing might be important regardless of tumor size. However, the true value of detecting these genetic alterations in FTCs and OTCs with limited capsular invasion only remains to be determined, and studies with longer follow-up are needed to investigate the prognostic implications of our findings.

## Data Availability

Datasets generated in this study will be available upon reasonable request.
